# Effectiveness of endoscopic gastric cancer screening in a rural area of Linzhou, China: results from a case–control study

**DOI:** 10.1002/cam4.812

**Published:** 2016-07-01

**Authors:** Qiong Chen, Liang Yu, Chang‐qing Hao, Jin‐wu Wang, Shu‐zheng Liu, Meng Zhang, Shao‐kai Zhang, Lan‐wei Guo, Pei‐liang Quan, Nan Zhao, Ya‐wei Zhang, Xi‐bin Sun

**Affiliations:** ^1^ Department of Cancer EpidemiologyHenan Cancer Hospital/InstituteAffiliated Cancer Hospital of Zhengzhou UniversityZhengzhou450008China; ^2^ Linzhou Cancer RegistryLinzhou Cancer HospitalLinzhou456500China; ^3^ Department of Environmental Health SciencesYale University School of Public Health60 College Street, LEPH 440New HavenConnecticut06520

**Keywords:** Case–control study, effectiveness, gastric cancer, screening program

## Abstract

In China, a large burden of gastric cancer has remained, and endoscopic screening was expected to reduce gastric cancer mortality. Therefore, a population‐based case–control study was conducted to evaluate the screening effect. The gastric cancer screening program was initiated in Linzhou in the year 2005, and endoscopic examination with indicative biopsy, for residents aged 40–69 years, was used to detect early cancer and precancerous lesion. In this study, cases were defined as individuals who had died of gastric cancer, which were selected from Linzhou Cancer Registry database. Controls were residents (six per case), who had not died of gastric cancer, from the same area as the case, and matched by gender and age (±2 years). The exposure status, whether cases and controls ever attended the screening or not, was acquired by inspecting the well‐documented screening records. Conditional logistic regression model was used to estimate the odds ratios (*OR*) and their 95% confidence intervals (95% *CI*). A total of 313 cases and 1876 controls were included in our analysis. Compared with subjects who never participated in screening, the overall *OR* for individuals who ever participated in screening was 0.72(95% *CI*: 0.54–0.97). The *OR* for lag time 4 years or longer was 0.68(95% *CI*: 0.47–0.98) and the *OR* for those who were aged 50–59 years were 0.56 (0.37–0.85). The results suggest a 28% reduction in risk of gastric cancer mortality by endoscopic screening, which may have significant implications for gastric cancer screening in rural areas of China.

## Introduction

Gastric cancer is the most common cancer in the digestive system. Worldwide, the world age‐standardized incidence and mortality were 12.1 and 8.9 per 100 000, which ranked fifth and third among cancers of all sites, respectively 1. Although the incidence and mortality in China has slightly decreased 2, high burden of gastric cancer still persists. The world standardized incidence and mortality of gastric cancer in China were 22.7 and 17.9 per 100 000, which ranked fourth and sixth in the world. A total of 404 000 incident cases and 325 000 deaths were estimated in 2012, which accounted for 43% and 45% of the world cases 1.

Early detection and diagnosis of gastric cancer during the routine medical services was difficult because patients were asymptomatic in the early cancer stages. Therefore, majority of the patients present themselves in the clinic only when the disease had progressed to an advanced stage; and hence, was related to bad survival. The observed 5‐year survival rate was 90% for early‐stage patients, while it was only about 10% for advanced‐stage patients 3. The proportion of early‐stage gastric cancers was less than 10% in China, and poor survival (the age‐standardized 5‐year relative survival: 27.4%) was observed by a population‐based study in China 4. Although the prognosis has improved over the past decades due to advances in diagnosis and treatment, gastric cancer is still among the lethal cancers. Therefore, early detection and diagnoses should be done to improve gastric cancer patients' prognosis.

Endoscopic screening for gastric cancer in moderate to high risk population was found to be cost effective 5, and it had been conducted in countries of East Asia with high gastric cancer incidence 6, 7, 8, 9. Gastric cancer deaths prevented by endoscopic screening were observed in a retrospective cohort study in Japan 10. Although positive results were observed in studies that evaluated the effect of gastric screening program, the effect remains unclear.

A national gastric cancer screening and early treatment program has been initiated since 2005 in rural and high gastric cancer incidence areas of China 11. The guideline for gastric cancer screening was made by China Cancer Research Foundation based on previous studies in the high risk areas 12. Treatment with precursor lesion and early stage gastric cancer was advised in the guideline to improve the gastric cancer prognosis 13, 14, 15.

Linzhou city (the former Linxian County) lies in the east side of the Taihang Mountains with a population of 1.05 million and is known for high incidence of esophageal and gastric cancer in the world. Therefore, Linzhou city was selected as a pilot area to conduct the population‐based screening program since 2005. Endoscopy with indicative biopsy for residents who were aged 40–69 years old were studied to identify cancer and precursor lesion. By 2015, more than 30 000 individuals aged 40–69 years old were screened in this program 16. In Linzhou, the population‐based Cancer Registry had been established since the 1960s, and the data quality had been evaluated by the criterion of International Agency for Research on Cancer (IARC) and the follow‐up data were used to estimate the survival of cancer patients of the population 4. In this study, the data of the cancer registry and the records of screening for gastric cancer were linked to analyse the effectiveness of the screening program in Linzhou, China.

## Material and Methods

The study was approved by the Institutional Review Boards at Henan Cancer Hospital and Linzhou Cancer Hospital. Informed consents were obtained from all participants prior to their enrollment according to the Declaration of Helsinki. All methods were carried out in accordance with the approved guidelines.

The gastric cancer screening program in Linzhou was organized and conducted by Linzhou Cancer Hospital. Residents lived in 124 villages of five towns (Hejian, Heshun, Dongyao, Hengshui, and Linqi) participated in the screening project for once at the time of 2005, 2008, 2011, 2012, or 2013. Subjects without history of diagnosed cancer and aged between 40 and 69 years old were eligible for the screening program, and 51.2% of the target population in these villages participated in the screening program. Endoscopy with indicative biopsy was used to detect and diagnose gastric cancer and precancerous lesions.

The endoscopic screening was carried out according to the guideline for esophageal and gastric cancer screening, early diagnosis, and treatment program 12. The participants were placed in the left lateral position, and the entire esophagus and stomach were visually examined by trained doctors. The stomach was stained with 0.2% indicarminum to help locate suspicious lesions, which were targeted for biopsy. Biopsy specimens were fixed in 10% buffered formalin, embedded in paraffin, cut into 5‐*μ*m sections, and stained with hematoxylin and eosin. The biopsy slides were read by two pathologists. Participants were recalled to the clinic when early lesions were histologically diagnosed, and intervention methods appropriate to the lesions' severity were used.

Information on participants' demographic characteristics, life style, and disease history were collected using a standardized questionnaire by trained doctors in Linzhou Cancer Hospital. The individual records of participants' information, endoscopy, and pathology reports were well documented and managed by the department of cancer epidemiology of Linzhou Cancer Hospital.

### Study population

All residents aged 40–69 years in these 124 villages, who did not express dissent to their records being used for evaluation purposes, were targeted as study population.

### Case selection

**Figure 1:**
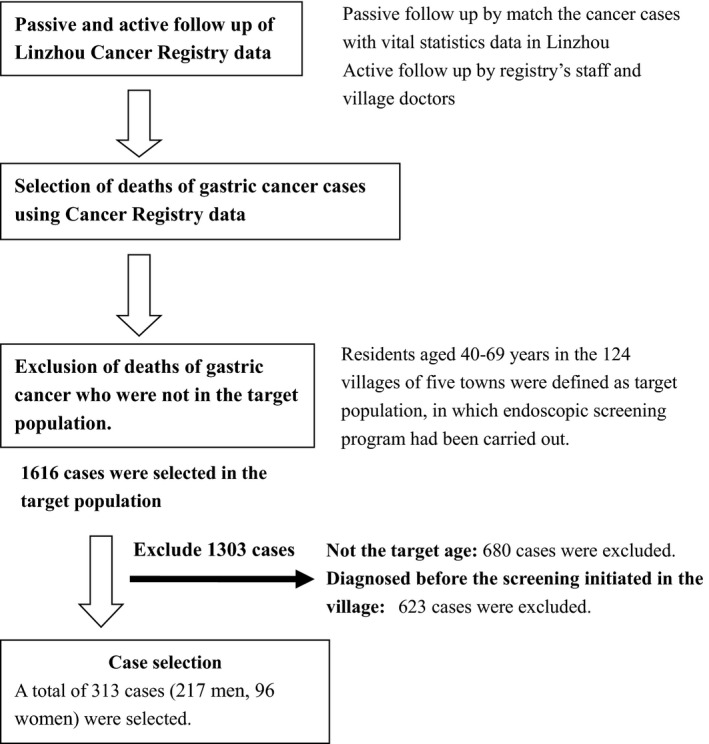
Flowchart for the selection of case subjects. Cases were defined as individuals who had died of gastric cancer in the 124 villages from September 2005 to December 2015. Cases were also required to meet the following criteria: 1) they must have been diagnosed as gastric cancer patients during the period of September 2005–December 2015; 2) the age at diagnosis with gastric cancer should be limited between 40 and 69 years old; 3) the diagnosis date must be later than the date of the screening program conducted; and 4) who were residents in the screening area.

The flowchart for selection of case subjects was shown in Figure 1. All the 124 villages have been covered by a population‐based Cancer Registry and the active and passive follow‐up procedure have been routinely performed by Registry's staff members and village doctors in Linzhou. Cases were selected from the database of Linzhou Cancer Registry, of which the quality had been evaluated by the criterion of IARC, and the data had been used in previous studies 4, 17. According to Morrison's method 18, cases were defined as individuals who had died of gastric cancer in the 124 villages from September 2005 to December 2015. Cases were also required to meet the following criteria: (1) they must have been diagnosed as gastric cancer patients during the period of September 2005–December 2015; (2) the age at diagnosis with gastric cancer should be limited between 40 and 69 years old; (3) the diagnosis date must be later than the date of the screening program conducted; and (4) who were residents in the screening area.

Based on the inclusion criterion, a total of 1616 deaths of gastric cancer had been identified. Among them, 680 patients were excluded due to their age being either less than 40 years old or over 69 years old when the screening started in their villages. Additional 623 cases were excluded due to the diagnosis date which was earlier than the date when the screening started in their residential villages. Finally, 313 cases were included into our final analysis.

### Control selection

Controls were selected from the resident roster which was generated from the well‐documented records of Resident Health Documents Program and New Rural Cooperative Medical Systems operated by the local government.

Deaths were excluded from the resident roster before the control selection procedure. Six controls for each case were randomly selected in the resident roster and were matched by age (±2 years), gender, and residence village. The controls must be alive at the time when their matched cases were diagnosed as gastric cancer. Even if the subjects were matched, individuals under 40 years of age were excluded because they had no opportunity to be screened. Two of the cases matched with five controls for each. Finally, 1876 controls were matched with the 313 cases.

### Exposure measurement

The beginning and end dates of screening in each village were extracted from the screening records. The prior screening status of the cases and controls was acquired by inspecting the screening records. The date of screening was also checked, and age was calculated at the time of screening for subjects who ever attended. The lag time was defined as the time between the date when the individual participated in screening test and 31 December 2014.

### Statistical analysis

Statistical analysis was conducted using SAS software (SAS Institute, Cary, NC). Chi‐square test was conducted to compare the difference of each characteristic between cases and controls. The odds ratios (ORs) and their 95% confidence intervals (95% CIs) were estimated using the conditional logistic regression models. The ORs were calculated to evaluate the associations between risk of gastric cancer mortality and endoscopic screening. Stratified analyses were performed by age and gender.

## Results

A total of 313 deceased gastric cancer cases (217 men, 96 women) and 1876 matched controls (1301 men, 575 women) were included in this study. The mean age of subjects being diagnosed with gastric cancer was 60.6 ± 6.6 years. There was no significant difference in diagnosis age between men (60.5 ± 6.8) and women (60.8 ± 6.2). **As** shown in Table**** 1 **,** approximately 50% of the cases were aged 50–59 years old. A total of 105 (33.5%) cases and 732 (39.0%) controls had ever participated in gastric cancer screening. Among the individuals who ever participated in the screening, majority of them were aged 50 years or older. The proportion of those who participated in screening was higher in controls than that in cases aged 50–59 years. A large proportion of individuals who participated in screening had lag time of 2 years or longer.

**Table 1:** Distribution of case and control subjects by gender and age

Screening factors	Men				Women				Total			
	Cases	%	Controls	%	Cases	%	Controls	%	Cases	%	Controls	%
Age												
40–49	32	14.8	181	13.9	10	10.4	64	11.1	42	13.4	245	13.1
50–59	95	43.8	588	45.2	50	52.1	305	53.0	145	46.3	893	47.6
60 years and over	90	41.5	532	40.9	36	37.5	206	35.8	126	40.3	738	39.3
Total	217	100.0	1301	100.0	96	100.0	575	100.0	313	100.0	1876	100.0
* χ* ^2^	0.19				0.12				0.18			
* P*	0.909				0.944				0.916			
Never screened	152	70.0	851	65.4	56	58.3	293	51.0	208	66.5	1144	61.0
Ever screened, stratified by age when participated in the screening												
40–49	5	2.3	37	2.8	4	4.2	35	6.1	9	2.9	72	3.8
50–59	25	11.5	212	16.3	24	25.0	174	30.3	49	15.7	386	20.6
60 years and over	35	16.1	201	15.5	12	12.5	73	12.7	47	15.0	274	14.6
* χ* ^2^	3.58				2.16				5.29			
*P*	0.312				0.541				0.153			
Ever screened, stratified by time duration since the date when participated in screening												
<2 years	5	2.3	24	1.8	3	3.1	16	2.8	8	2.6	40	2.1
2–3 years	15	6.9	109	8.4	9	9.4	70	12.2	24	7.7	179	9.5
>4 years	45	20.7	317	24.4	28	29.2	196	34.1	73	23.3	513	27.4
* χ* ^2^	2.40				2.04				4.24			
* P*	0.508				0.564				0.245			

The subjects who ever participated in endoscopic screening were stratified by the age (40–49 years, 50–59 years, 60 years or older) when they participated in screening, lag time (<2 years, 2–3 years, 4 years and more) since the date when they participated in screening.

Compared with individuals who never participated in the screening, the OR of endoscopic screening for individuals who ever participated in screening program was 0.72 (95% CI: 0.54–0.97). The reduced risk of gastric cancer mortality was associated with the screening program with lag time period equal to or longer than 4 years (OR (95% CI):0.68(0.47–0.98)). The risk of lag time less than 4 years was not statistically significantly; the trend analysis was statistically significant (*P* for trend: 0.022).

When examining the association between endoscopic screening and risk of gastric cancer mortality by age of subjects when they participated in screening program, the reduced risk of gastric cancer mortality was only observed in 50–59‐year‐old individuals (OR = 0.56, 95% CI: 0.37–0.85). Stratified by anatomic site (cardia gastric and noncardia/site not specified gastric), statistically significant association (OR 0.49, 95% CI: 0.28–0.84) was found in 50–59‐year old subjects for cardia gastric. (Table 2).

**Table 2:** Odds ratio of death from gastric cancer for screened subjects compared with never screened subjects

Screening factors	Total						Cardia						Noncardia/site not specified					
	Cases	%	Controls	%	OR	95% CI	Cases	%	Controls	%	OR	95% CI	Cases	%	Controls	%	OR	95% CI
Ever attended																		
Never	208	66.5	1144	61.0	1		124	63.6	679	58.1	1		84	71.2	465	65.7	1	
Ever	105	33.5	732	39.0	0.72	0.54–0.97	71	36.4	489	41.9	0.73	0.51–1.05	34	28.8	243	34.3	0.71	0.43–1.16
Lag Time since the screening																		
Never screened	208	66.5	1144	61.0	1		124	63.6	679	58.1	1		84	71.2	465	65.7	1	
<2 years	8	2.6	40	2.1	1.32	0.51–3.45	3	1.5	21	1.8	0.8	0.19–3.35	5	4.2	19	2.7	2.10	0.56–8.06
2–3 years	24	7.7	179	9.5	0.70	0.41–1.18	15	7.7	112	9.6	0.69	0.36–1.35	9	7.6	67	9.5	0.70	0.29–1.69
4 years and more	73	23.3	513	27.4	0.68	0.47–0.98	53	27.2	356	30.5	0.74	0.47–1.17	20	17	157	22.2	0.56	0.29–1.08
*P* for trend					0.022						0.106						0.091	
Age of screened subjects when they participated in screening																		
Never screened	208	66.5	1144	61.0	1		124	63.6	679	58.1	1		84	71.2	465	65.7	1	
40–49 years	9	2.9	72	3.8	0.56	0.23–1.40	7	3.6	46	3.9	0.76	0.23–2.53	2	1.7	26	3.7	0.37	0.08–1.75
50–59 years	49	15.7	386	20.6	0.56	0.37–0.85	30	15.4	250	21.4	0.49	0.28–0.84	19	16.1	136	19.2	0.69	0.36–1.33
60 years and over	47	15.0	274	14.6	1.00	0.66–1.54	34	17.4	193	16.5	1.05	0.63–1.74	13	11	81	11.4	0.90	0.41–1.98
*P* for trend					0.128						0.216						0.374	

The subjects who ever participated in endoscopic screening were stratified by the age (40–49 years, 50–59 years, 60 years or older) when they participated in screening, lag time (<2 years, 2–3 years, 4 years and more) since the date when they participated in screening.

Stratified analysis by gender was shown in Table 3. The ORs of endoscopic screening for men and women were 0.75(95% CI: 0.53–1.08) and 0.67(95% CI: 0.40–1.10). Statistically significant association was only observed in 50–59‐year‐old, screened male subjects (OR 0.55, 95% CI: 0.32–0.93).

**Table 3:** Odds ratio of death from gastric cancer for screened subjects compared with never screened subjects stratified by gender

Screening factors	Men						Women					
	Cases	%	Controls	%	OR	95% CI	Cases	%	Controls	%	OR	95% CI
Ever attended												
Never	152	70.1	851	65.4	1		56	58.3	293	51.0	1	
Ever	65	29.9	450	34.6	0.75	0.53–1.08	40	41.7	282	49.0	0.67	0.40–1.10
Lag Time since the screening												
Never screened	152	70.1	851	65.4	1		56	58.3	293	51.0	1	
<2 years	5	2.3	24	1.8	1.43	0.41–4.96	3	3.1	16	2.8	1.19	0.26–5.34
2–3 years	15	6.9	109	8.4	0.75	0.40–1.43	9	9.4	70	12.2	0.59	0.23–1.50
4 years and more	45	20.7	317	24.4	0.70	0.44–1.10	28	29.2	196	34.1	0.64	0.34–1.22
*P* for trend					0.086						0.098	
Age of screened subjects when they participated in screening												
Never screened	152	70.1	851	65.4	1		56	58.3	293	51.0	1	
40–49 years	5	2.3	37	2.8	0.70	0.22–2.21	4	4.2	35	6.1	0.42	0.10–1.72
50–59 years	25	11.5	212	16.3	0.55	0.32–0.93	24	25.0	174	30.3	0.58	0.30–1.15
60 years and over	35	16.1	201	15.5	1.03	0.63–1.70	12	12.5	73	12.7	0.94	0.42–2.10
*P* for trend					0.270						0.236	

The subjects who ever participated in endoscopic screening were stratified by the age (40–49 years, 50–59 years, 60 years or older) when they participated in screening, lag time (<2 years, 2–3 years, 4 years and more) since the date when they participated in screening.

## Discussion

Due to the high burden of gastric cancer, countries such as Japan and Korea in East Asia had undertaken nationwide population‐based gastric cancer screening strategy to reduce the incidence and mortality 6, 19, 20. Studies have been conducted to evaluate the effect after the program had been conducted for several decades; however, the effect of the screening program as a routine practice remains unclear 20. In China, the screening programs for gastric cancer have been conducted in areas with high gastric cancer incidence, which are mainly in rural areas.

Case–control study has been regarded as an appropriate method to evaluate the efficacy of widespread cancer screening programs 18, 21, 22. Our study reported a 28% reduction in risk of gastric cancer mortality in subjects who participated in endoscopic screening compared with individuals who never participated in any screening. The endoscopic screening for gastric cancer has been conducted as a national program in Korea 6, and has been partly adopted in population‐based screening in Japan 8. The effectiveness of endoscopic screening remains unclear 23, 24, 25, although positive results have been reported 7, 8. Our result was consistent with a previous case–control study in Japan 26, which reported a 30% reduction in the risk for gastric cancer mortality 26. The effect of endoscopic screening would be affected by several factors, such as sample size, screening rate, and so on, in which the skills of endoscopist and availability of gastroscope were thought to be pivotal. Although endoscopy is widely available in major cities in China, the availability and accessibility in rural areas are limited 20, 27. Furthermore, the effect of endoscopic submucosal dissection of early gastric cancer highly depends on the skills of endoscopists 13, which needs to be improved in rural areas of China.

When stratified analysis by anatomical sites was conducted, statistically significant effect was not observed both in cardia gastric and noncardia gastric. Although endoscopy specialists have been trained to detect precursor lesions or early stage cancer of cardia gastric by the guideline in previous several years when the screening program initiated, noncardia gastric precursor lesions were also detected in the actual endoscopic procedure. When the results were stratified by anatomy site, approximately 30% reduction in gastric cancer mortality was observed both in cardia gastric and noncardia gastric; however, it was not statistically significant, maybe due to a small sample size.

Our study found a 32% reduction in the risk of gastric cancer mortality among individuals who had undergone a screening test 4 years before or even longer as compared to individuals who never had endoscopic screening. However, there was no statistically significant reduction in risk of gastric cancer mortality among individuals who had been screened less than 4 years. The OR for lag time less than 2 years were higher than those for two‐three years and 4 years and more but not statistically significant, it may partly due to the small number of cases within the group. It was also possible that lag time was too short to be able to conclude the endoscopic screening effect in less than 4 years lag time group. Previous studies on breast cancer and colorectal cancer indicated that several years were needed to show the effect after introduction of screening program 28. Results from a screening effect evaluation study in Korea also showed that the strongest effect was observed in 24–35 months 29.

Endoscopic screening was found to benefit 50–59‐year‐old individuals in our study; however, statistically significant effect was not observed in women by stratified analysis. We did not find statistically significant association with screening in 40–49‐year‐old subjects and 60 years or older subjects, which may be partly due to the small sample size and low compliance.

In our study, the screening histories for cases and controls were acquired by investigating the well‐documented records of screening program, and the recall bias was believed to be eliminated. However, certain limitations should also be considered when interpreting the study results. Firstly, information on socioeconomic status, education, and income were not obtained, which were known to be related with self‐selection bias. Although Duffy et al. suggest a method for correcting for noncompliance bias in case–control studies to evaluate cancer‐screening programs 30, we could not adjust this bias due to the lack of previously published randomized data as adjusting factor for gastric cancer.

Secondly, due to the small number of cases, the screening effect in subgroups was not observed. Our study only included data from Linzhou city. The screening program was also conducted in several other cities in Henan Province, however, these cities were not included in our study due to the lag time was too short to assess the effect. Thirdly, subjects who died of gastric cancer within 2 years after the screening initiated in the village were also included in the final analysis. There is a potential bias that it may magnify the screening effect.

To the best of our best knowledge, this is the first population‐based case–control study to evaluate the effect of endoscopic screening on gastric cancer mortality in China. Our results suggested a 28% reduction in gastric cancer mortality. It may have significant implications for gastric cancer screening in China, especially in rural areas. However, prudent interpretation is needed due to potential limitations and future well‐designed studies with larger sample size are needed to confirm our study results.

## Conflicts of Interest

No potential conflicts of interest were disclosed.
